# Functional characterization of a yellow laccase from *Leucoagaricus gongylophorus*

**DOI:** 10.1186/s40064-015-1464-y

**Published:** 2015-10-30

**Authors:** Priscila Tomie Leme Ike, Ariele C. Moreira, Fernando G. de Almeida, Douglas Ferreira, Willian Garcia Birolli, Andre Luiz Meleiro Porto, Dulce Helena F. Souza

**Affiliations:** Departamento de Química, Universidade Federal de São Carlos, São Carlos, SP Brazil; Instituto de Química de São Carlos, Universidade de São Paulo, São Carlos, Brazil

**Keywords:** Yellow laccase, *Leucoagaricus gongylophorus*, Kinetic enzyme, Non-phenolic substrate, Molecular modeling

## Abstract

In this work we have identified, using mass spectrometry, two laccases produced by *Leucoagaricus gongylophorus*. One of them, Lac1Lg, was isolated, purified and characterized. Lac1Lg, a monomeric enzyme, was studied using ABTS and syringaldazine substrates. Lac1Lg presented kcat/Km almost threefold higher for syringaldazine than for ABTS, showing a higher catalytic efficiency of Lac1Lg for syringaldazine. The interference of several metal ions and substances in the laccase activity were evaluated. Lac1Lg did not absorb at 600 nm, which is a characteristic of so-called yellow laccases. Lac1Lg also was able to oxidize non-phenolic substrate (anthracene) in the absence of an exogenous mediator, showing that the enzyme has potential to explore in biotechnological processes. Our Lac1Lg three-dimensional molecular model, constructed using homology modeling, showed that the Lac1Lg catalytic site is very closed to blue laccases.

## Background

Laccases (EC 1.10.3.2, *p*-diphenol:dioxygen oxidoreductase) are enzymes that can oxidize a wide spectrum of aromatic and non-aromatic compounds (Gianfreda et al. 1999) and contain copper atoms in the catalytic center being usually called multicopper oxidases (Baldrian 2006; Piontek et al. 2002). These enzymes are widely distributed in higher plants, fungi, bacteria and insects, presenting different physiological function (Riva 2006; Wu et al. 2013). Fungal laccases generally present high redox potential giving them potential for application in technological processes (Brijwani et al. 2010) such as in bioremediation of some toxic chemical wastes, as well as in the production of ethanol and food, the paper and textile industries, and organic synthesis (Viswanath et al. 2014; Thurston 1994).

Laccases have been described as containing four copper atoms, which are classified into three different types (T1, T2 and T3), depending on the region where they are found in the enzyme molecule (Solomon et al. 1996). The T1 copper site is the site that binds the primary reducing substrate and T2 and T3 sites (containing copper types-2, 3 and 4), where reduction of molecular oxygen and release of water takes place. Laccases with typical UV–visible spectra (at resting state) show two maxima around 280 and 600 nm and one shoulder near 330 nm (Madhavi and Lele 2009). The maximum at 600 nm has been attributed to type 1 copper (T1), which is responsible for the intense blue color of the laccases, and the weak absorbance near 330 nm has been related to type 3 copper (T3). These copper atoms have been also classified in three groups using electron paramagnetic resonance (EPR) spectroscopy: types 1 and 2 copper (T1 and T2) are EPR detectables and type 3 copper (T3) has no EPR signal (Leontievsky et al. 1997). Although these features have been reported for most studied laccases, some laccases do not have an absorption band around 600 nm (showing also no EPR signal due the type 1 copper) and are called ‘yellow’ or ‘yellow/white’ laccases (Leontievsky et al. 1997). However, more recently a yellow laccase isolated from the fungus *Sclerotinia sclerotiorum* showed no absorbance at 600 nm but it was detectable in the EPR spectrum making this enzyme different of others yellow laccases (Mot et al. 2012).

Typical blue laccases require the presence of small compounds (the so-called chemical mediators) to catalyze the reaction where the substrates are non-phenolic compounds (Bourbonnais and Paice 1992) but Leontievsky et al. (1997) have described that yellow laccases catalyze reactions using non-phenolic substrates without mediators. These behaviors of yellow laccases have been associated with modifications in the type 1 copper, for example, with the binding of a lignin-derived mediator in the catalytic site (Leontievsky et al. 1997). This mediator could be coming from the culture medium of which the enzyme has been isolated. However, Huang et al. (2011) described a yellow laccase obtained in a recombinant form (i.e. in a synthetic medium) and this suggests that lignin decomposition products are not required for the existence of yellow laccases. Sequence alignments of laccases have shown that there is no obvious pattern corresponding to yellow or blue laccases (Daroch et al. 2014). The latest hypothesis is that small changes in the sequences of yellow laccases can be responsible for the formation of polyproline helices and alternative folding of these enzymes (Daroch et al. 2014; Mate et al. 2013).

*Leucoagaricus gongylophorus,* a basidiomycete, is a fungus that lives in symbiosis with leaf-cutting ants *Atta* and *Acromyrmex*, which are considered major herbivores in the tropics (Cherret 1986). The metabolic potential of *L. gongylophorus* has been analyzed and has been demonstrated that the fungus metabolizes several different polysaccharides (Gomes de Siqueira et al. 1998; Silva et al. 2006). As part of our efforts to understand the enzymes involved in the polysaccharide metabolic process, we started the studies of the enzymes involved in this pathway (Moreira et al. 2014). Recently, the draft genome *L. gongylophorus* was sequenced (Accession: PRJNA179280) and studies have showed that this fungus produces distinct sets of lignocellulases throughout the different stages of biomass degradation, including numerous cellulases and laccases that likely play an important role in lignocellulose degradation (Aylward et al. 2013). Nine putative laccase-coding genes in the genome of *L. gongylophorus* have also been reported, and it was observed that laccases detoxify secondary plant compounds mediating the nutritional association between leaf-cutting ants and fungus-garden symbionts (De Fine Licht et al. 2013).

In this work, two laccases from *L. gongylophorus* were identified; one of them was isolated, purified, biochemically and kinetically characterized, and identified by mass spectrometry. This laccase was a yellow laccase lacking absorbance at 600 nm and oxidizing non-phenolic substrate in the absence of exogenous mediators. To our knowledge, no three-dimensional structure for a yellow laccase is known. Next, we built a structural model for this laccase based on homology modeling, which allowed us to analyze the catalytic site, including the amino acids that can be involved in the catalysis.

## Results and discussion

### Laccases production

The production of extracellular laccases by *L. gongylophorus* was monitored by assaying for activity in the culture medium with the method of ABTS every 48 h for 15 days. No significant difference in activity measured in the two culture media, with and without veratryl alcohol, was observed, which indicates that the laccase activity is not induced by the presence of the alcohol. However, we kept the veratryl alcohol in the culture medium due to its ability to inhibit protease activity (Meza et al. 2007). Laccase activity in the extracellular medium using veratryl alcohol was measured over 15 days; we observed a growth curve up to a maximum activity on the 11th day. The production of fungal laccases depends on the species and strain of the fungi and frequently occurs in 5- and 14-day-old cultures (Elisashvili and Kachlishvili 2009).

### Laccase purification and characterization

**Fig. 1:**
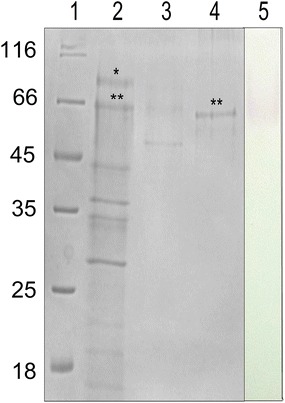
Purification and activity of laccase from *L. gongylophorus* (Lac1Lg). SDS–PAGE analysis in a 15 % gel stained with Comassie. *Lane 1* molecular weight markers; *markers* used were β-galactosidase (116 kDa), bovine serum albumin (66.2 kDa), ovalbumin (45.0 kDa), lactate dehydrogenase (35.0 kDa), Rease Bsp98I (25.0 kDa), β-lactoglobulin (18.4 kDa); *lane 2* crude extract applied onto hydroxyapatite column; *lanes 3* and *4* fractions with laccase activity eluted from hydroxyapatite and superose 12 columns, respectively; *lane 5* zymography of sample of the *lane 4*. *Asterisks* (*) represents Lac2Lg and (**) represents Lac1Lg

After detecting laccase activity in the culture medium, the crude extract was fractionated by ionic exchange chromatography on a hydroxyapatite column. Fractions with laccase activity were pooled, dialyzed and applied onto a size-exclusion column. Fractions with laccase activity eluted from the column were pooled and concentrated. The purified laccase, here called Lac1Lg, presents an apparent molecular weight of about 66 kDa as analyzed in SDS-PAGE (Fig. 1) and the sample purity was estimated in about 67 % by the Image Lab™ Software (BioRad).

Compared with standard protein applied onto the superose 12 column, the Lac1Lg showed to be a monomeric enzyme. Most fungal laccases are monomeric proteins, but several laccases have been described as exhibiting a dimeric or trimeric structure (Baldrian 2006) and even tetrameric laccase has been reported (Thurston 1994).

The fraction containing Lac1Lg purified from superose 12 column was analyzed with zymography demonstrating laccase activity. Table 1 summarizes the yield of the enzyme in the purification procedure using syringaldazine substrate.

**Table 1:** Recovery of activities fractions after purification of Lac1Lg using syringaldazine substrate

	Protein concentration (µg mL^−1^)	Total activity (mU)	Specific Activity (mU mg^−1^)	Recovery (%)	Purification fold
Crude extract	300	0.4	275	100	1
Hydroxyapatite I	25	0.1	907	8.3	3.3
Superose 12	1	0.06	5716	0.3	20.8

Here we have observed that Lac1Lg presents a light yellow color and also has no significant absorbance at 600 nm. To better understand the characteristics of Lac1Lg, we performed a reaction with a non-phenolic substrate (anthracene) and Lac1Lg enzyme in both the presence and the absence of ABTS, a well-known exogenous mediator. In both reactions, we detected, through mass spectrometry, the oxidation of the anthracene producing anthraquinone. Our results show that Lac1Lg is a yellow laccase and that it does not need chemical mediators to catalyze reactions with non-phenolic substrates, which is an excellent characteristic for its use in biotechnological processes.

### Effect of pH and temperature on laccase activity and stability

**Fig. 2:**
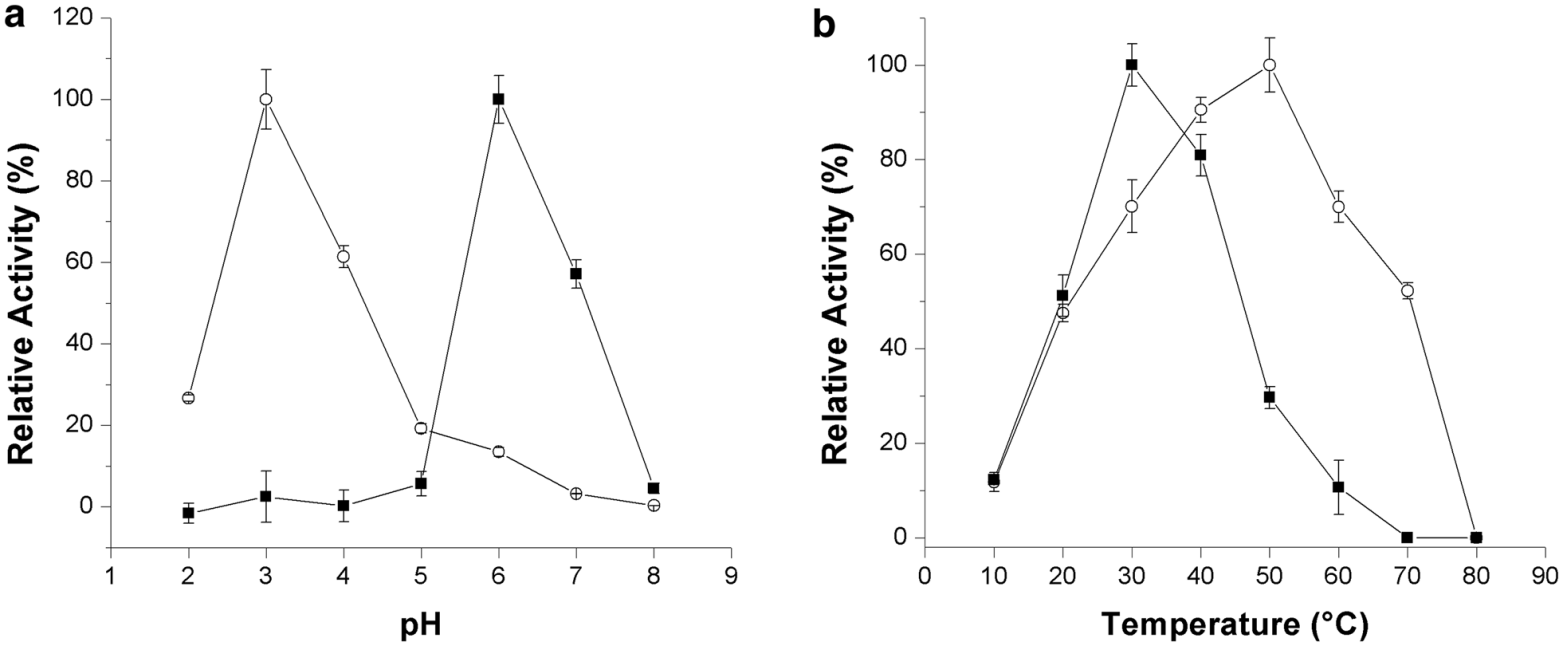
Analysis of the influence of pH (**a**) and temperature (**b**) on Lac1Lg using syringaldazine (*filled square*) and ABTS (*unfilled circle*) substrates. Assay conditions: McIlvaine buffer for pH 2.0–8.0

The purified Lac1Lg had its activity studied using syringaldazine and ABTS as substrates at different pH. The maximum activity observed for Lac1Lg was at pH 6.0 and pH 3.0, for syringaldazine and ABTS, respectively (Fig. 2a). The optimum pH values found for Lac1Lg are in agreement with the literature, which describes the pH optima of laccases as highly dependent on the substrate. For phenols, the fungal laccases often present an optimal pH that can range from 3 to 7; for non-phenolic substrates, the optimal pH is <4 (Madhavi and Lele 2009). Recent studies showed two yellow laccases from the basidiomycete fungus *Stropharia aeruginosa* with optimum pH values of 4.0 and 3.0 for syringaldazine and ABTS, respectively (Daroch et al. 2014). Yellow laccases from *Trametes hirsula* and *Aspergillus niger* showed maximum activity at pH 2.4 and pH 2.2 for ABTS, respectively (Haibo et al. 2009; Tamayo-Ramos et al. 2012). Yellow laccase from *Pleurotus ostreatus* D1 showed optimum pH values of 7.0 and 4.0 for syringaldazine and ABTS, respectively (Pozdnyakova et al. 2006). For ABTS substrate a yellow laccase isolated from *Lentinus squarrosulus* presented maximum activity at pH 4.5 (Mukhopadhyay and Banerjee 2014).

To study the interference of temperature in laccase activity of Lac1Lg, we have analyzed the reaction at different temperature values using the buffer with an optimal pH value. Using syringaldazine, Lac1Lg presents activity at an optimum temperature of 30 °C (Fig. 2b) and using ABTS, Lac1Lg presents the highest activity at 50 °C (Fig. 2b). The interference of temperature in laccase activity varies greatly from one strain to another, and optimum temperature values have been found for fungal laccases spanning a range of 25–80 °C, with most enzymes having maximum activity at 50–70 °C (Madhavi and Lele 2009). For example, the yellow laccase isolated from *Lentinus squarrosulus* presented optimum temperature at 40 °C with ABTS (Mukhopadhyay and Banerjee 2014) and the yellow laccase from *Sclerotinia sclerotiorum* had optimal activity within the range of 60–70 °C with the same substrate (Mot et al. 2012).

The thermal stability of Lac1Lg was verified by incubating the enzyme in the optimal pH optimum at different temperatures and after the incubation period, syringaldazine was added and the activity was measured. Lac1Lg retained about 35 and 17 % activity after being incubated for 1 h at 30 and 40 °C, respectively. At 50 °C, <10 % of the enzyme activity was retained after 15 min of incubation.

### The effect of some substances in the activity of laccase

Table 2 shows the influence of some substances in the activity of Lac1Lg. The laccase activity is dependent upon the presence of copper ions; the addition of EDTA, which is a chelating agent, was expected to eliminate activity. Using EDTA 5 mM the Lac1Lg residual activity was only about 28 % and it was almost abolished with EDTA 10 mM. The presence of the SDS detergent almost completely inhibited the activity of Lac1Lg, indicating that this substance negatively changes the structure of the enzyme; the same occurs for laccase produced by the hyphomycete *Chalara* (syn. *Thielaviopsis*) *paradoxa* (Robles et al. 2002). However, for many laccases, SDS does not affect enzyme activity, or even enhance the reaction at low concentration, such as the laccase produced by *Fusarium solani* MAS2 (Wu et al. 2010). Lac1Lg activity was almost completely inhibited by 2-β-mercaptoethanol, which is a strong reducing agent, indicating the presence of disulfide bridges in its structure.

**Table 2:** Effects of various compounds on laccase activity of the purified Lac1Lg

Compound	Residual activity (%)
Control	100 ± 2.2
CuSO_4_ (2 mM)	91.7 ± 0.4
CuSO_4_ (20 mM)	36.6 ± 1.1
EDTA (1 mM)	81.5 ± 4.8
EDTA (5 mM)	28.1 ± 2.8
EDTA (10 mM)	2.9 ± 0.3
SDS (5 mM)	2.8 ± 0.8
2-β-mercaptoethanol (10 mM)	0.1 ± 0.2
NaCl (20 mM)	62.7 ± 1.3
NaCl (50 mM)	31.8 ± 1.1
NaF (0.1 mM)	80.4 ± 2.4
NaF (1 mM)	76.6 ± 7
NaF (5 mM)	0
CuSO_4_ (2 mM)	91.7 ± 0.4
CuSO_4_ (20 mM)	36.6 ± 1.1
CrCl_3_ (2 mM)	88.3 ± 4.4
CrCl_3_ (20 mM)	0
FeCl_3_ (2 mM)	58.7 ± 7.90
FeCl_3_ (20 mM)	0
CoCl_2_ (2 mM)	96.4 ± 1.9
CoCl_2_ (20 mM)	72.3 ± 4.4
NiCl_2_ (2 mM)	94.8 ± 0.5
NiCl_2_ (20 mM)	44.7 ± 1.7
ZnCl_2_ (2 mM)	98.2 ± 12.3
ZnCl_2_ (20 mM)	17.4 ± 2.7
DMSO 1 %	83.4 ± 12.1
DMSO 10 %	50.5 ± 5.3

The literature describes how Cl^−^ can inhibit laccase activity, and previous studies of laccases have showed I_50_ values of 0.05–600 mM (Xu 1996). Therefore, laccase inhibition by halides is peculiar for each laccase. Lac1Lg had its activity decreased about 37 and 23 % in the presence of 20 mM of NaCl and 1 mM NaF, respectively.

The activity for many laccases has no influence or is increased by the presence of Cu^2+^; however, as found for Lac II isolated from *Trametes versicolor* (Lorenzo et al. 2005), Lac1Lg was inhibited in the presence of 20 mM Cu^2+^. For other metal ions, such as Cr^3+^, Fe^3+^, Ni^2+^, Mn^2+^ and Zn^2+^, Lac1Lg activity was decreased with increasing concentration of ions. Co^2+^ had the mildest effect on the enzyme activity and Fe^3+^ was the most aggressive. Lac1Lg showed considerable resistance in the presence of DMSO, which is very useful in assays of hydrophobic substrates.

### Kinetic parameters

The kinetic parameters Km, V_max_, kcat, and kcat/Km were determined for the Lac1Lg enzyme (Table 3) under optimal conditions of pH and temperature utilizing syringaldazine and ABTS as substrates. Therefore, using McIlvaine buffer, the assays were carried out in pH 6.0 and at 30 °C for syringaldazine and pH 3.0 and at 50 °C for ABTS. The lower Km value for Lac1Lg was with syringaldazine, which indicates a higher affinity for this substrate than for ABTS.

**Table 3:** Michaelis–Menten kinetic constants of Lac1Lg

Substrate	Km (μM)	V_max_ (μM min^−1^)	kcat (s^−1^)	kcat/Km (s^−1^ μM^−1^)
ABTS	131.9	3.02	110.7	0.84
Syringaldazine	5.1	0.16	11.7	2.3

Lac1Lg presents kcat/Km almost threefold higher for syringaldazine than for ABTS, showing a higher catalytic efficiency of Lac1Lg for syringaldazine.

Despite other yellow laccases that present Km values for oxidation of syringaldazine and ABTS lower than that obtained for Lac1Lg (Leontievsky et al. 1997), Pozdnyakova et al. (2006) described a yellow laccase from the fungus *Pleurotusostreatus* with Km values close to Lac1Lg (8.7 µM for syringaldazine and 110 µM for ABTS) and Mukhopadhyay and Banerjee (2014) described a yellow laccase with Km and V_max_ of 71.4 µM and 9.1 µM min^−1^ for ABTS.

### Protein identification by MS/MS database search

**Fig. 3:**
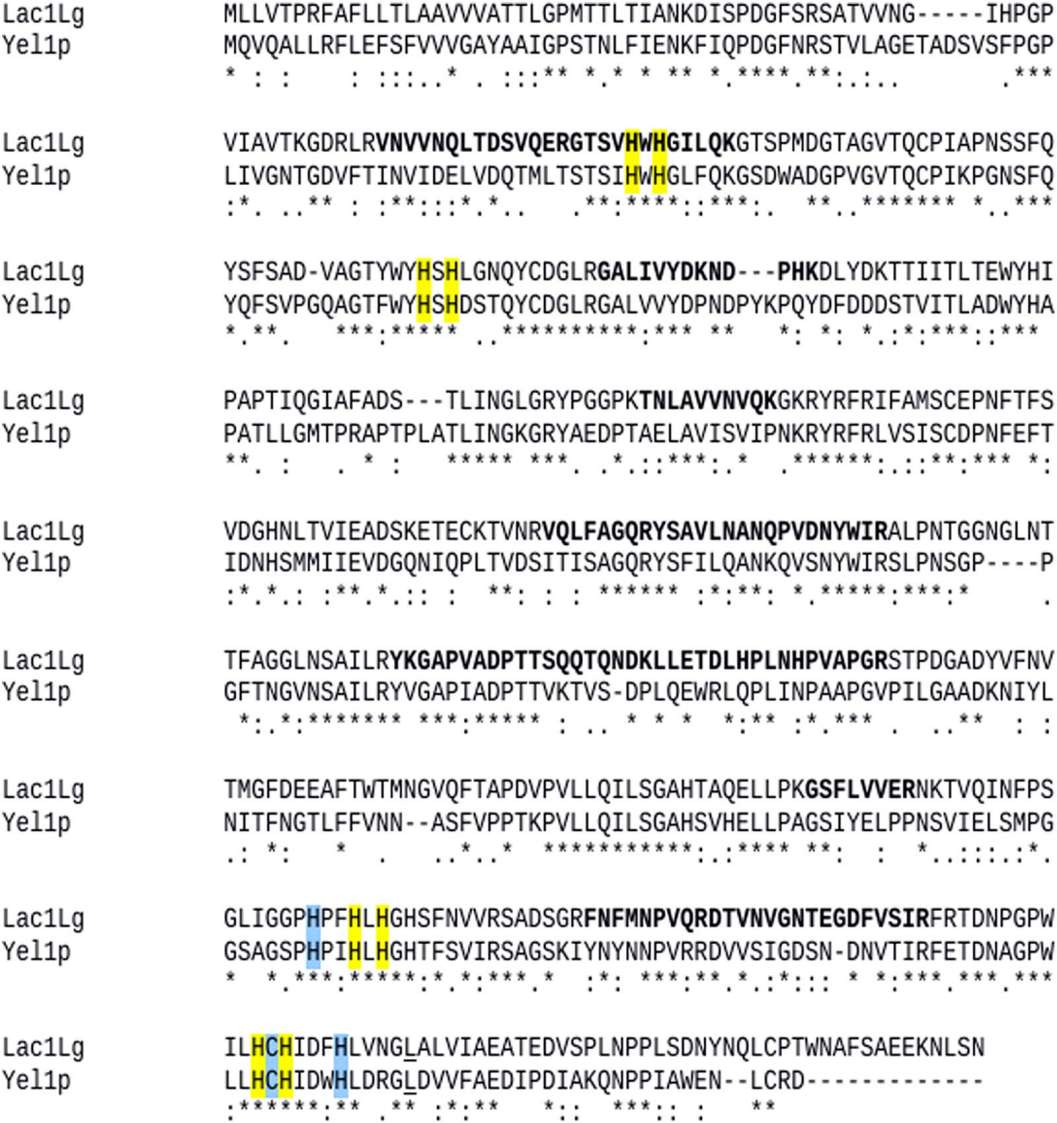
Sequential alignment of yellow laccases Lac1Lg and Yel1p (Ducros, et al. 1998) using ClustalW2 program (McWilliam et al. 2013). *Asterisk* (*) represents identical residue in both sequences, *colon* represent high similarity residues in the sequences and *dot* represent residues with medium similarity. In *yellow* histidines which could bind to type-2,3 and 4; in *blue* amino acids which could bind to copper type-1. Leucine residue close to Cu type-1 is *underlined*

To sequentially identify the Lac1Lg, the sample was applied to a 1D SDS-PAGE gel; next, the band was excised from the gel, treated with trypsin and the peptides were analyzed by on line LC–MS nanoflow. Databases with different numbers of sequences were used to increase the protein identification confidence. By searching the nucleotide and protein databases, we identified a laccase sequence (accession number NCBI 409151740) in the published sequence of *L. gongylophorus* strain Ae322 so-called LgLcc6 (De Fine Licht et al. 2013). The analysis showed 13 peptides with about 27 % of sequence coverage (Table 4). The Lac1Lg (corresponding to LgLcc6) sequence is shown in Fig. 3 and the peptides are in bold in the sequence. This figure shows also a sequential alignment of Lca1Lg and a yellow laccase from *Stropharia aeruginos*a (discussed below).

**Table 4:** Peptide coverage sequences of first hit to enzyme identified in gel band

Search	Accession number	Identified protein name (organism)	Sequence coverage (%)	Peptide coverage sequences
Leucoagaricus_NCBI_sequest	409151740	Laccase (*L. gongylophorus*)	27.34	YSAVLNANQPVDNYWIR YKGAPVADPTTSQQTQNDK VNVVNQLTDSVQER DTVNVGNTEGDFVSIR GAPVADPTTSQQTQNDK LLETDLHPLNHPVAPGR TNLAVVNVQK FNFmNPVQR GSFLVVER VQLFAGQR GALIVYDK GTSVHWHGILQK GALIVYDKNDPHK

The LgLcc6 calculated molecular weight is 57,070 Da and the Lac1Lg was observed in SDS-PAGE with about 66,000 Da. This difference in the molecular weight may be due to glycosylation, which is quite common in laccases (Hakulinen et al. 2002) and often assigned to be responsible for the thermal stability, proteolytic susceptibility, copper retention (Christensen and Kepp 2013). Laccases have been described with typically 3–10 glycosylation sites, predicted from their amino acid sequence (Rodgers et al. 2010 that represents 10 and 25 %, but laccases with 65–80 % of saccharides have been described (Gianfreda et al. 1999). Prediction of glycosylation sites for Lac1Lg shows potential *O*-Linked and *N*-Linked glycosylated sites. Furthermore, the enzyme sample was bound in Concanavalin A (Con A) Sepharose affinity resin and eluted with α-d-mannopyranoside (data not shown).

### Molecular modeling of Lac1Lg

Crystallographic studies have contributed to better understanding of the mechanism and function of laccases. It has been observed that the four regions containing copper ions and their ligands are quite conserved in the laccase structures studied (Piontek et al. 2002). There are eight histidines at the copper binding cluster T2/T3 and two histidines, one cysteine and one methionine in a axial position at the copper binding site T1 acting as ligands for copper (Xu et al. 1999). However, most fungal laccases have leucine (Leu) or phenylalanine (Phe) at the position corresponding to the axial methionine; these amino acids do not coordinate with the copper that remains a tri-coordinate T1 site (Ducros et al. 1998). The several mutagen studies developed to understand the relationship between the deficiency in the coordination of type 1 copper and the electron transfer function of the T1 site show that the lack of an axial ligand might be responsible for the high E° observed in fungal laccases (Xu et al. 1999).

**Fig. 4:**
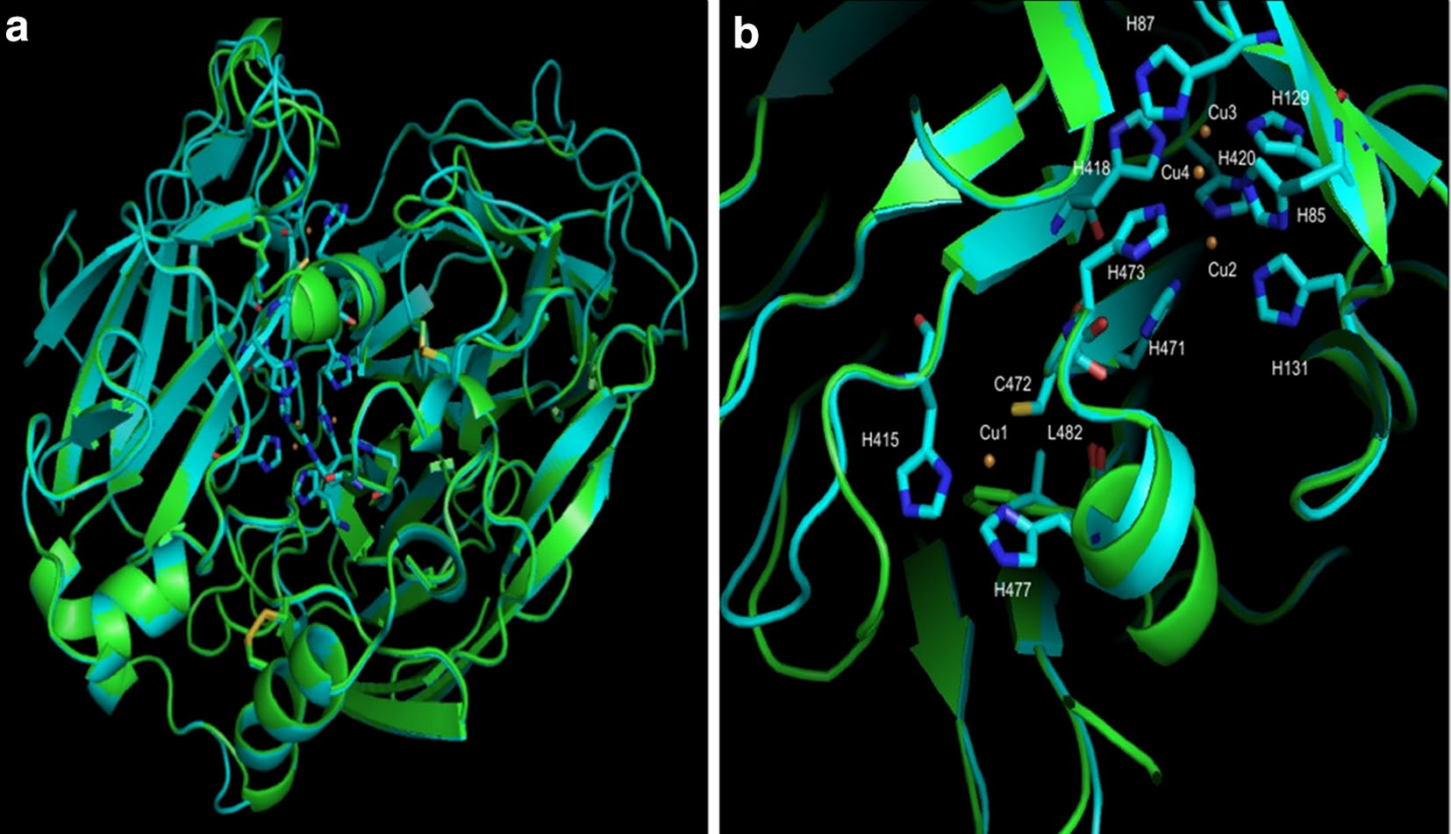
Three-dimensional model of Lac1Lg constructed by molecular modeling using SWISS-MODEL program (Biasini et al. 2014) and TvL 3D structure as template molecule (PDB 1gyc.1). Copper ions from the TvL 3D structure are draw as orange spheres. **a** General view of the model and the structure superposition. In *blue*, molecular model for Lac1Lg and in *green* the 3D structure for TvL. **b** Copper type-1 region (T1 site) and copper T2/T3 sites. Only His from Lac1Lg model are showed in the T2/T3 sites

There is no three-dimensional structure known of the yellow laccases; therefore, to better understand this class of enzyme, the three-dimensional structure of the Lac1Lg enzyme was modeled. The 3D structure (PDB 1gyc.1) of a blue laccase from the fungus *Trametes versicolor* (TvL enzyme), which has approximately 55 % identity to Lac1Lg, was used as a template molecule. Figure 4a shows the general view of the superposition of the three-dimensional structure of TvL (green) and the model of Lac1Lg (blue). The model presents a good fit with the structure (RMSD of 1.14Å as calculated with *SuperPose* web *server*—http://wishart.biology.ualberta.ca/SuperPose/), and we observed that the different regions are mainly in the loops. TvL and Lac1Lg have five cysteine (cys) residues with two disulfide bridges (shown in orange in Fig. 4a) and one cys binding the copper type 1 (T1 site). We can also observe the cavity in the upper part of Fig. 3a where copper type 1 is found and where the substrate bind is expected (Piontek et al. 2002). Figure 4b shows, in more detail, the copper type 1, along with the two histidines and the cysteine that can bind the copper. TvL has not the fourth binder for copper type 1 (because methionine was replaced by Phe, represented in green in Fig. 4b) and Lac1Lg has a Leu in this same position (in blue in Fig. 4b). Studies have shown that the substitution of methionine for amino acids that do not bind to copper contributes to the higher redox potential of some laccases (Piontek et al. 2002). Figure 4b shows also the T2/T3 site with the three copper ions, and we can observe the eight histidine residues that are expected to bind the copper types 2, 3 and 4.

The superposition of the molecular model and the structure suggested that TvL (a blue laccase) and Lac1Lg (a yellow laccase) are probably structurally conserved, as observed in the blue laccases. This study showed also that Lac1Lg has the amino acids required for binding of the four copper ions. Sequence alignment (Fig. 3) between Lac1Lg and Yel1p (GenBank: AFE48785.1), a yellow laccase from the basidiomycete fungus *Stropharia aeruginos*a (Daroch et al. 2014) showed that they share 50 % amino acid identity. We can identify the two histidines and one cysteine that could bind to type 1 copper (in blue in Fig. 4b); replacing the axial methionine is a leucine (underlined in Fig. 3). The eight histidines that bind to copper ions of the T2/T3 site were identified in the Lac1Lg sequence (in yellow in Fig. 3). Recently, Daroch et al. (2014) analyzed several sequences of blue and yellow laccases and did not identify any amino acid motifs that could explain the differences between the yellow and blue laccases. The results presented here indicated that the overall folding of the enzymes (blue and yellow laccases) are very similar and that the active site region is also highly conserved.

### Identification of another laccase from *L. gongylophorus*

The band shown in Fig. 2 with an apparent molecular weight greater than 66 kDa (here called Lac2Lg) was also analyzed by mass spectrometry and was identified a laccase sequence (AFV15785.1) called LgLcc1, with 12 % of sequence coverage. De Fine Licht et al. (2013) developed an interesting study on the laccase activity in the fungus garden of the *Acromyrmex echinatior* cutting ant. The authors identified nine laccase genes in *L. gongylophorus* (called LgLcc1–LgLcc9) and, by analysis of gene expression, detected that LgLcc1 is the most expressed gene. They suggested the function of detoxification of secondary plant compounds for laccases from *L. gongylophorus*. They also showed that heterologous LgLacc1 expressed in yeast showed laccase activity.

In summary, we have identified two laccases from *L. gongylophorus* whose genes were annotated in the genome of the fungus; one of them was characterized as a yellow laccase.

## Conclusions

We have identified, in fungal culture conditions described, two laccases produced by *L.gongylophorus*. Lac1Lg and Lac2Lg were identified by mass spectrometry. Lac1Lg had optimal temperature and pH, as well as kinetic parameters, determined using both ABTS and syringaldazine substrates. Lac1Lg does not absorb at 600 nm and is able to oxidize non-phenolic substrates in the absence of exogenous mediators, allowing us to classify it with the so-called yellow laccases. Sequences alignment between blue and yellow laccases have shown amino acids conserved in both classes of laccases and here we have shown that these amino acids are in a favorable position for binding the copper ions.

## Methods

### Strain and reagents

2,2′-Azino-bis(3-ethylbenzothiazoline-6-sulfonic acid) diammonium salt (ABTS), syringaldazine and liver bovine catalase were purchased from Sigma Chemical Co., St. Louis. All other chemicals were of analytical grade. *L. gongylophorus* strain FF-2006 was isolated from a lab nest of *Atta sexdens rubropilosa* and was maintained in 2 % malt extract, 0.5 % bacteriological peptone, 0.2 % yeast extract, and 2 % agar.

### Enzymatic assay

Enzymatic activity was determined by oxidation of ABTS (Wolfenden and Wilson 1982), a general substrate for some oxidases and by oxidation of syringaldazine, a specific substrate for laccase (Harkin et al. 1974). The mixture reaction for ABTS assay contained 0.5 mM ABTS, 20 mM sodium acetate buffer (pH 5.0), suitable amount of culture medium and 1 U of catalase in a total volume of 0.5 mL. Catalase was added to remove any residual H_2_O_2_. This reaction was incubated for 10 min at room temperature. Oxidation of ABTS was spectrophotometrically monitored at 420 nm using the extinction coefficient of oxidized ABTS of 36,000 M^−1^ cm^−1^.

The mixture reaction for syringaldazine assay contained 10 μM syringaldazine, 20 mM sodium acetate buffer (pH 6.0), 10 μL of enzymatic solution in a total volume of 0.5 mL. Oxidation of syringaldazine was measured at 525 nm with an extinction coefficient of 65,000 M^−1^ cm^−1^. All assays were performed in triplicate.

One unit of laccase activity was defined as the amount of enzyme needed to oxidize 1 μmol of substrate per minute.

### Laccases production

To produce laccases, the mycelium of approximately 5 cm disks from 60-day-old cultures cultivated on solid medium was transferred to a 500 mL Erlenmeyer flask with 100 mL of modified Tien and Kirk (1988) medium (without dimethyl succinate). This inoculum was kept at room temperature (25 °C) without shaking for 15 days. To determine the best medium for express laccases, this experiment was also realized in this culture medium in the absence of veratryl alcohol.

To determine the optimal time of culture, aliquots of 0.5 mL were collected every 48 h for monitoring enzymatic activity by oxidation of ABTS. After determining the optimum time of cultivation to produce laccases, the culture medium was filtered through a 0.22 μm membrane and then concentrated by lyophilization, re-suspended in water (in 10 % of initial volume) and stored at −20 °C. This filtered medium was called enzymatic extract.

### Laccase purification

Enzymatic extract (10 mL) was dialyzed against 10 mM sodium phosphate pH 5.8 plus 0.2 mM CaCl_2_ (buffer A) for 12 h, and was applied to an ionic exchange chromatography on a hydroxyapatite column (1.5 cm × 12 cm, CHTTM Ceramic hydroxyapatite type I—Bio-Rad), previously equilibrated with buffer A in an AKTA-FPLC™ system (GE Healthcare Sciences). Elution was carried out using buffer B (400 mM sodium phosphate pH 5.8 plus 0.0075 mM CaCl_2_) in a 30-cv (column volume) linear gradient (0–100 % of buffer B) at a flow rate of 1.0 mL min^−1^, and the eluted fractions were tested for laccase activity using syringaldazine as substrate. Fractions with laccase activity were pooled, dialyzed against buffer C (50 mM sodium phosphate pH 7.0) and concentrated (10×) using ultrafiltration system (Amicon Ultra-15 Millipore). This sample was purified onto a size-exclusion chromatography on a superose 12 10/300 GL column (GE Healthcare Life Sciences) previously equilibrated with buffer C that was supplemented with 150 mM NaCl. The isocratic elution was performed with the same buffer at a flow rate of 0.3 mL min^−1^.

All purification steps were performed at 20 °C and were followed by enzymatic assays and by sodium dodecyl sulfate polyacrylamide gel electrophoresis (SDS-PAGE).

### UV–vis absorbance spectra

As a part of the characterization of the physicochemical properties of the purified laccase, its absorbance spectrum from 200 to 800 nm was obtained in a UV–vis Beckman Coulter spectrophotometer.

### Zymography analysis

For rapid detection of laccase activity, we utilized zymography technique with sodium dodecyl sulfate—polyacrylamide gel electrophoresis (SDS-PAGE) (Gonçalves and Steiner 1996). The samples loaded into the gel were not boiled and, after running, the polyacrylamide gel was renatured by incubation in a 50 mM sodium acetate buffer pH 6.0, for 1 h (4 washes of 15 min) at room temperature. Then, the activity was revealed with a solution of 10 µM syringaldazine in the same buffer.

### Concentration and molecular weight determination

Protein concentration was determined using Coomassie Brilliant Blue G250 according to the Bradford method using the Bio-Rad protein assay kit (Bio-Rad Laboratories, Richmond, CA) and bovine serum albumin (BSA) as standard.

Molecular weight of the purified enzyme was estimated by both SDS-PAGE and gel-filtration on a column of superose 12 calibrated by elution of standard protein BSA.

### Determination of optimum temperature and pH

To evaluate the pH and temperature effects on enzyme activity, the assays were developed using syringaldazine (10 µM) and ABTS (1 mM) as substrates and the concentration of Lac1Lg was 0.02 and 0.03 µg mL^−1^, respectively.

To evaluate the pH interference the assays were performed in McIlvaine buffer at a pH of 2.0–8.0. To evaluate the influence of temperature on enzyme activity, the enzymatic assays were performed in the optimum pH buffer in different incubation temperatures (10–70 °C).

### Thermal stability

Thermal stability was verified by incubating the enzyme at optimal pH buffer in different temperatures (30–70 °C) for a period of 15 min to 2 h. After the incubation period, the syringaldazine substrate was added to measure enzyme activity.

### Kinetic characterization

The kinetic parameters were determined for purified laccase at the optimal pH and temperature by measuring the initial reaction velocity and varying the substrate concentration from 0.5 to 20 μM for syringaldazine and from 0.1 to 2 mM for ABTS. The experiments were performed in triplicate, and the Michaelis–Menten constant (Km) and maximum reaction (V_max_) values were calculated with GraphPad Prism 5.0 software.

### Interference of some substances in the laccase activity

The effects of the presence of some substances on the enzyme activity were determined by incubating the enzyme with the substance for 10 min at optimal pH and temperature before performing the assay activity. Activity was measured using syringaldazine as substrate and compared with the enzyme activity as measured in the absence of substances (control).

### Mass spectrometry analysis

The mass spectrometry analysis of the isolated laccase (Lac1Lg) was carried out in gel-tryptic digestion. Sample was applied in polyacrylamide gel electrophoresis (SDS-PAGE) and the protein bands excised from the gel were tryptically cleaved according to the method published by Shevchenko et al. (1996). Before LC–MS/MS analysis, samples were desalted using ZipTips^®^ and kept at −20 °C until the LC–MS/MS analysis.

#### LC–MS/MS analysis

Peptides were analyzed by online nanoflow LC–MS on an EASY-nLC II system (Thermo Scientific) connected to an LTQ-Orbitrap Velos instrument (Thermo Scientific) via a Proxeon nanoelectrospray ion source. Peptides were separated on an analytical EASY-Column (10 cm, ID75, 3 µm, C18-Thermo Scientific) previously trapped in a pre-column EASY-Column (2 cm, ID100, 5 µm, C18-Thermo Scientific). Tryptic digested peptides were separated using a 60 min linear gradient from 0 to 60 % acetonitrile containing 0.1 % formic acid at 300 nL min^−1^ flow rate.

The LTQ-Orbitrap Velos mass spectrometer was operated in positive ion mode using DDA (data-dependent acquisition) mode, in which the 20th most intense precursor ions from a full MS scan were selected for fragmentation by collision-induced dissociation (CID). Full MS scans were performed with 60,000 full-width half-maximum (FWHM) nominal resolution settings and the m/z range for MS scans was 400–1200. The normalized CID collision energy was 35 eV for a doubly charged precursor ion of 2 m/z, activation Q of 0.250 and activation time of 10 ms. The minimum signal threshold was 15,000 counts and, for dynamic exclusion, it was considered 1 repeat count with a duration of 30 s. In order to discriminate the charge state of the peptides, the charge state screening was enabled and ions with either unassigned charge state or a single charge were rejected. The instrument was calibrated externally according to the manufacturer’s instructions.

#### Database search

The MS/MS spectra from each LC–MS/MS run were searched against five different databases with two different search engines and in-house Proteome Discoverer 1.4 software (Thermo, USA). MS/MS ions search criteria were used as follows: full tryptic specificity was required, two missed cleavage sites was allowed, carbamidomethylation (C) was set as fixed modification and the oxidation (M) was set as the variable modification, precursor ion mass tolerances were set at 10 ppm for all MS acquired in an Orbitrap mass analyzer, and the fragment ion mass tolerance was set at ±0.6 Da for all MS2 spectra acquired.

Two analytical LC–MS/MS replicates were performed for protein excised from the gel and the raw files were considered for protein database search. The databases were downloaded by typing “Leucoagaricus” as a keyword on both NCBI and Uniprot sites, as well as “Laccase”. The “Fungi_NCBI” database was used directly from the MASCOT 2.2.4 search engine with NCBInr filtered by Fungi taxonomy. The result of each run was filtered with the peptide confidence value set to high to obtain an FDR of <1 % at the peptide level. At the protein level, the minimum number of peptides is 1 for the protein, count only ranking 1 peptide and a peptide count only in the top-scored proteins were applied for all data filtration.

### Molecular modeling

Protein sequence alignments were done using ClustalW2 program (McWilliam et al. 2013). The sequence of LgLcc6 (AFV15790.1) was used as an input file for the SWISS-MODEL program (Biasini et al. 2014) in the construction of the three-dimensional model for the Lac1Lg enzyme. The Pymol program (The PyMOL Molecular Graphics System, Version 1.2r3pre, Schrödinger, LLC) was used to visualize the model and to superpose the model and the three-dimensional structure of TvL, a blue laccase from the fungus *Trametes versicolor* (PDB 1gyc.1).
